# Dynamic metabolic regulation of histone modifications during the yeast metabolic cycle

**DOI:** 10.1371/journal.pone.0323242

**Published:** 2025-05-20

**Authors:** Bárbara Guzmán-Dinamarca, Raúl Conejeros, Marcelo Rivas-Astroza

**Affiliations:** 1 Universidad Tecnológica Metropolitana, Departamento de Biotecnología, Santiago, Chile; 2 Pontificia Universidad Católica de Valparaíso, Escuela de Ingeniería Bioquímica, Valparaíso, Chile; Texas A&M University, UNITED STATES OF AMERICA

## Abstract

Eukaryotes achieve a wide range of stable phenotypes by virtue of epigenetic modifications. However, what drives epigenetic diversification in the first place remains an open question. Here, we investigated the dynamic interplay between the production fluxes of epigenetic cosubstrates and histone post-translation modifications (PTMs) in *Saccharomyces cerevisiae*’s Yeast Metabolic Cycle (YMC). We developed a novel approach integrating flux analysis with transcriptomic data to investigate the production fluxes of acetyl-CoA and SAM and their influence on histone marks H3K9Ac and H3K4me3. Our results show that acetyl-CoA and SAM flux dynamics are asynchronous during the YMC, suggesting distinct regulatory roles. Gene ontology analysis revealed that genes whose enrichment of H3K9Ac correlates with acetyl-CoA dynamics are associated with metabolic functions, while genes whose enrichment of H3K4me3 correlates with SAM dynamics are associated with translation processes. Finally, we found evidence that chromatin accessibility on genes promoter regions was a precondition for the metabolic fluxes influencing the enrichment of H3K4me3 and H3K9Ac. These findings support the concept that metabolism provides timely cosubstrates essential for histone PTMs.

## Author summary

We investigated the dynamic interplay between metabolism and epigenetic modifications in yeast cells. Epigenetic modifications are like decorations on DNA that can change how genes are expressed, and they play a crucial role in determining cell identity. While the importance of these modifications is well-known, what triggers their changes remains unclear. We focused on two key epigenetic modifications, H3K9Ac and H3K4me3, and their connection to the metabolic molecules acetyl-CoA and SAM, respectively. By developing a new computational approach, we found that the production of these metabolic molecules fluctuates during the yeast metabolic cycle, suggesting they have distinct regulatory roles. Our analysis revealed that genes associated with acetyl-CoA are primarily involved in metabolic functions, while genes associated with SAM are linked to cell cycle regulation and protein synthesis. Additionally, we found that the accessibility of DNA plays a crucial role in determining which genes are modified. Our findings shed light on the intricate relationship between metabolism and epigenetic modifications, offering potential implications for understanding broader biological processes like cell differentiation and cancer development.

## Introduction

The intricate relationship between the epigenome and cellular metabolism is at the frontier of our biomedical [1–3] and industrial [4] research efforts, offering potential breakthroughs in these fields. Central to this interplay are histone post-translational modifications (PTMs), such as acetylation, methylation, and phosphorylation. These PTMs are key epigenetic modulators of gene expression, DNA accessibility, and alternative splicing [5]. The cellular metabolism is deeply intertwined with these context-specific epigenetic modifications, providing cosubstrates like acetyl-CoA, α-ketoglutarate, and S-adenosyl methionine (SAM) for the enzymes catalyzing histones PTMs [6–8]. However, accurately quantifying metabolic fluxes, particularly those generating PTMs cosubstrates, remains a significant challenge [9]. To bridge this gap, computational models inferring metabolic fluxes have been fundamental to advance our comprehension of the complex epigenetic-metabolic nexus. Still, many questions remain open.

Epigenetic enzymes responsible for adding or removing PTMs on histone tails are sensitive to the levels of specific metabolites, such as SAM, acetyl-CoA, and NAD+ [10–12]. For example, acetyl-CoA acts as substrate for histone acetyltransferases, which catalyze histone acetylation [13,14]. Histone methyltransferases utilize SAM to add methyl groups on histone tails [15,16]. The dynamic changes in specific histone modifications, such as H3K9 acetylation (H3K9Ac) and H3K4 trimethylation (H4K4me3), are of particular interest due to their established roles as cellular regulators of gene expression and metabolic state [17–21]. H3K9Ac is typically found at gene regulatory elements [17] and mediates switching from transcription initiation to elongation [22], whereas H3K4me3 is commonly enriched at the transcription starting site of actively expressed genes [23,24]. Correlation studies suggest a link with between the enrichment of H3K9Ac and H3K4me3, and the abundance of their metabolic co-substrates. In yeast, for instance, acetyl-CoA levels correlate with changes in gene expression [13,25]. Similarly, SAM levels have been linked to lifespan, stress response alterations [26] and diverse gene expression patterns [27,28], further highlighting the intricate connection between metabolism and chromatin modification [29]. However, a clear understanding of why certain genomic regions become enriched with specific histone PTMs while others do not remains elusive. One hypothesis suggests that chromatin accessibility is a prerequisite for histone modifications, with open chromatin regions being more susceptible to the influence of acetyl-CoA and SAM concentrations in the cytosol [30,31]. Testing this hypothesis directly is challenging due to the current inability to precisely measure the production fluxes of acetyl-CoA and SAM. The distribution of metabolic fluxes within the cell can be measured for only a dozens of reactions, typically those involved in glycolysis and the Krebs’ cycle. To estimate fluxes far removed from the intake pathways, including those related to the production of histone PTM cosubstrates, it is indispensable the use of computational models of the cellular metabolism.

Constraint-based models (CBMs) have been instrumental in estimating the distribution of cellular metabolic fluxes (the fluxome) [32]. These models utilize linear constraints derived from metabolite mass balances and flux bounds to define the solution space of possible fluxomes [33–35]. Flux balance analysis (FBA), the most influential CBM, selects the fluxome that maximizes a linear combination of fluxes representing the biomass growth rate [36]. FBA has been extended to incorporate context-specific information (e.g., transcriptomics or measured exchange fluxes) to tailor its estimations for specific phenotypes [37,38], a capacity that has been exploited to study epigenome-metabolism interactions [39]. In particular, FBA-based methods have been used to estimate the production flux of acetyl-CoA by using a multi-objective function where biomass growth rate is maximized along the production flux of acetyl-CoA [40,41]. In principle this methodology can be extended to consider the production flux of additional epigenetic cosubstrates. However, this would require to add user-defined weighting parameters for each extra objective. Thus, the study of the effect of multiple epigenetic cosubstrates on the epigenetic landscape remains an active area of research.

Alternatively, there are CBMs maximizing the fluxome’s Shannon entropy [35] which offer a strictly convex objective function, ensuring a unique optimal solution. By being rooted in the principle of maximum entropy, these CBMs predict the fluxomes that can happen in the greatest number of ways and the least biased. This is evidenced by the higher prediction performance of this type of CBM over FBA-based ones on a variety of conditions for bacteria, yeast, and human metabolic networks [35,42]. These entropy-based CBMs can also incorporate transcriptomic information [42], allowing for phenotype-specific inferences of metabolic fluxes without the need to predefined the relative weights of cosubstrate fluxes. This makes this type of CBM particularly suitable to study the effect of various epigenetic cosubstrates on the landscape of histone PTMs.

Here, we used data-driven workflow to analyzed various libraries of transcriptomics (RNA-seq), histone PTMs (ChIP-seq), cellular intake fluxes of oxygen, and biomass growth rates [25] as well as chromatin accessibility (ATAC-seq) [43] to study how the metabolic state influences the epigenetic landscape. We used as a study case the yeast metabolic cycle (YMC) of *Saccharomyces cerevisiae* as it exhibits consistent metabolic cycles under glucose-limited conditions where gene expression, metabolites, and histone PTMs significantly fluctuate [25,44,45]. Thus, *S. cerevisiae*’s YMC provides an informative system to study the metabolic-epigenetic interactions [46–48]. Using our data-drive process, we uncovered surprising complementary roles of the production fluxes of acetyl-CoA and SAM on acetylation and methylation of histones. Complemented with ATAC-seq data [43] we found that chromatin accessibility was a precondition for this cosubstrates to effectively affect the histones. By combining various data sets, our approach offers a comprehensive view of the effects of multiple histone PTMs cosubstrates on chromatin modifications.

## Materials and methods

### RNA-seq, ChIP-seq, and ATAC-seq sources

We used Kuang *et al*. [25]’s dataset, which includes gene expression and histone changes sampled at 16 time points during one oscillation of the YMC. Some of the transcriptomic and epigenetic libraries were not sampled at equivalent times, for example, the RNA-seq of reductive building phase has 7 sampling points, whereas ChIP-seq has just 6. To create a synchronized sequence of ChIP-seq and RNA-seq sampling points, we used Sanchez *et al*. methodology [18] where oxygen consumption levels were used as an identifier of cell metabolic states. As a result, Kuang *et al*. [25]’s RNA-seq libraries reported at time points 10 and 11, as well as their ChIP-seq libraries reported at time points 13 and 14, were averaged. This resulted in a data set of 15 matching time points between ChIP-seq and RNA-seq (S1 Fig).

We used Gowans *et al*. [43] dataset to assess chromatin accessibility. This dataset consist on ATAC-seq libraries sampled at 6 time-points during one oscillation of the YMC. Two samples were taken at the OX stage, roughly at the center of the first and second half of this stage. In the same manner, two samples were taken at RB stage, and another two at RC stage.

### Enrichment of epigenetic marks

We used information from genome annotations of the *S. cerevisiae* (SacCer2, downloaded from UCSC Database http://genome.ucsc.edu/), and the ChIP-seq read counts reported by Kuang *et al*. [25] to compute the enrichment of H3K4me3 and H3K9Ac marks on gene promoter regions (+/- 500bp around transcription starting site).

### Genome-scale metabolic model

We used the high-quality, manually-curated genome-scale metabolic model iMM904 [49] downloaded from the BiGG Models database [50]. It contains a description of 1577 biochemical reactions, 1226 metabolites and 905 genes of the metabolism of *S. cerevisiae*. To make the predictions of acetyl-CoA and SAM fluxes in *S. cerevisiae* we added to the genome-scale metabolic model iMM904 [49] the reactions associated with acetylation and methylation of histones. We included 6 reactions directly related to acetylation (S1 Table) and 10 reactions associated with methylation (S2 Table). This reconstruction in terms of acetylation is given by the reaction catalyzed by ATP citrate lyase and the necessary metabolites to carry out this reaction. It was taken into account that acetyl-CoA and the substrates for the synthesis of acetyl-CoA can diffuse between the cytosol and the nucleus through the nuclear pore. Therefore, the flux through the protein acetylation reaction is representative of acetylation changes in both cytosol and nuclear proteins [40]. In relation to methylation, we added exchange reactions for metabolites that are part of the methionine cycle and necessary for methylation to occur. Glycogen and trehalose are the two glucose reserves of yeast cells [51]. Therefore, we verified the existence of these sugars in the iMM904 model, finding that only the trehalose reaction was present, therefore we also added the glycogen reaction.

### Data analysis workflow

In our data analysis workflow we employed an entropy-based CBM approach [42] to estimate the network-scale distribution of metabolic fluxes at each of the 15 time points within the YMC. By integrating time-specific transcriptomic data, oxygen uptake rates, and biomass growth rates into the CBM, we generated 15 fluxomes spanning the entire YMC. From these fluxomes, we extracted the time-varying production fluxes of acetyl-CoA and SAM. In the next sections are presented: the details of the calculation for time-specific oxygen consumption fluxes; and the mathematical description of the CBM conditioned on transcriptomic, biomass growth rate, and oxygen uptake rates data.

#### Oxygen uptake and biomass growth rates dynamics over the *S. cerevisiae*’s YMC.

**Fig 1:**
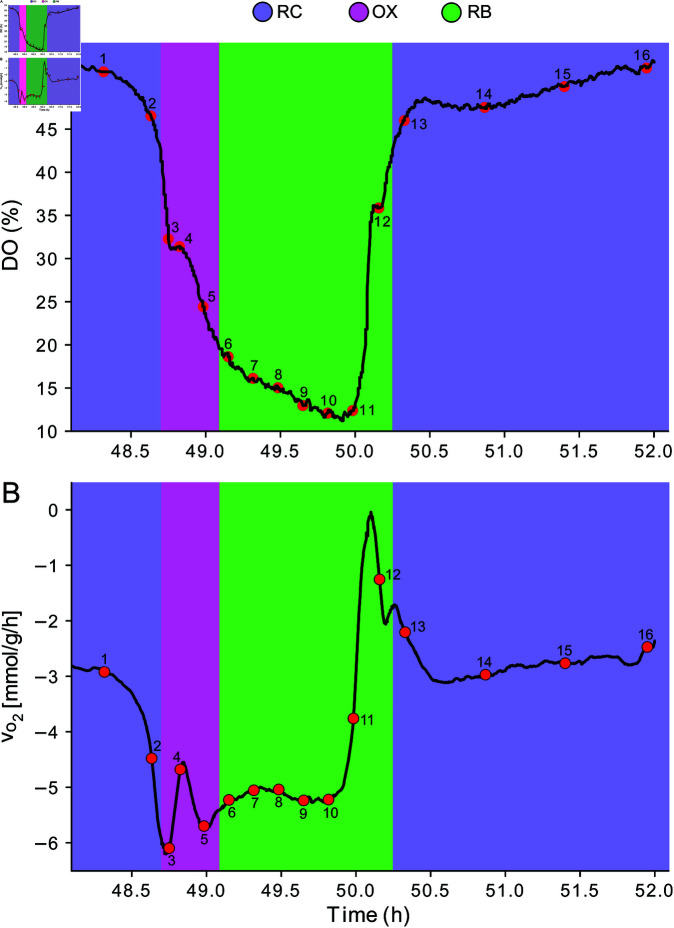
Oxygen oscillations in YMC. We used the percentages of dissolved oxygen reported by Kuang [25] (A) to compute the uptake rates of oxygen at 16 time-points of the YMC (B). Metabolic phases are color-coded as follows: magenta, OX phase; green, RB phase; blue, RC phase. Negative flux values indicates oxygen consumption.

We computed the oxygen uptake rates based on the fraction of dissolved oxygen (*DO*) reported by Kuang *et al*. [25] (see Fig 1). We modeled the mass balance of the temporal oxygen concentration variation in the culture media as:

$$\frac{dc}{dt}=k_{L}a\cdot (c^{*}-c)-q_{O_{2}}\cdot X$$ (1)

where ${\text{c}}^{*}$ is the saturation concentration of oxygen [mmol ${\text{L}}^{-1}$], *c* the actual oxygen concentration [mmol ${\text{L}}^{-1}$], *k*_*L*_*a* is the volumetric oxygen transfer coefficient [${\text{h}}^{-1}$], $q_{O_{2}}$ is the specific oxygen uptake rate [mmol ${\text{g}}^{-1}\;{\text{h}}^{-1}$], and *X* is the biomass concentration [${\text{g L}}^{-1}$]. As DO is reported by Kuang *et al*. [25] as a fraction of ${\text{c}}^{*}$, Eq. 1 was rewritten in terms of *DO* = *c*/c^*^, leading to an oxygen mass balance based on the dissolved oxygen fraction:

$$c^{*}\cdot \frac{dDO}{dt}=k_{L}a\cdot (c^{*}-DO\cdot c^{*})-q_{O_{2}}\cdot X$$ (2)

Simplifying by ${\text{c}}^{*}$:

$$\frac{dDO}{dt}=k_{L}a\cdot (1-DO)-q_{O_{2}}\cdot \frac{X}{c^{*}}$$ (3)

$$q_{O_{2}}=\frac{c^{*}}{X}\cdot \left[k_{L}a\cdot (1-DO)-\frac{dDO}{dt}\right]$$ (4)

We calculated the *dDO*/*dt* values using the Savitzky-Golay method of order 2 [52] with the help of Python’s derivative library dxdt function. We used *k*_*L*_*a* = 225 [${\text{h}}^{-1}$], based on the data reported by Paciello and Parascandola [53] for a reactor configuration similar to the one used by Kuang *et al*. [25]. This enabled us to obtain the oxygen exchange flux, $v_{O_{2}}=-q_{O_{2}}$, for each time (Fig 1B). Additionally, we used 0.1 [${\text{h}}^{-1}$] as the biomass growth rate as this was the dilution rate, which was the value reported by Kuang *et al*. [25].

#### Constraint-based model of cellular metabolism.

All CBMs estimate the distribution of metabolic fluxes by constraining the solution space as follows. In a metabolic network with *N* reactions and *M* metabolites, the rates of changes in metabolite concentrations, $\dot{m}\in {\mathbb{R}}^{M}$, are expressed as:

$$Sv=\dot{m}$$ (5)

where the term $v\in {\mathbb{R}}_{+}^{N}$ represents the set of metabolic fluxes, while $S\in {\mathbb{R}}^{M}$ × ${\mathbb{R}}^{N}$ is the stoichiometric matrix. The thermodynamic potentials define the direction of the reactions and are expressed as lower bounds ($LB\in {\mathbb{R}}^{N}+$) and upper bounds ($UB\in {\mathbb{R}}^{N}+$) for the fluxes of the reactions. All fluxes are considered positive, with reversible reactions represented as the sum of a forward ($v_{i}^{f}$) and reverse ($v_{i}^{r}$) flux. Under the assumption of a steady-state, equation 5 reduces to Sv=0. The thermodynamic potentials that determine the reaction directions are integrated in the form of lower ($LB\in {\mathbb{R}}^{N}+$) and upper ($UB\in {\mathbb{R}}^{N}+$) bounds. All these constraints define the fluxome space:

$$𝒫=\{v\in {\mathbb{R}}_{+}^{N}|Sv=0,LB\leq v\leq UB\}$$ (6)

Different CBMs differ on how a fluxome is selected from this space. We use the selection method proposed by González *et al*. [42], who chooses the v∈𝒫 that produces the highest Shannon entropy [54] given and experimentally observed transcriptome, $g^{T}=[g_{1},...,g_{N}]$, where *g*_*i*_ is the gene expression value of the complex encoding the enzyme catalyzing reaction *i*. González *et al*. [42] show that the Shannon entropy, $H_{g}(v)$, can be expressed as:

$$H_{g}(v)=-\sum_{i=1}^{N}\frac{v_{i}}{\sum_{j}v_{j}}\log\left(\frac{v_{i}/\sum_{j}v_{j}}{g_{i}/\sum_{j}g_{j}}\right)$$ (7)

By maximizing $H_{g}(v)$, this approach predicts the most likely metabolic flux distribution with minimal bias and has demonstrated superior predictive performance compared to alternative models [35]. Consequently, we selected *v* by solving the following strictly convex optimization problem:

$$\max_{v}H_{g}(v)\;\text{subject to:}\;v\;\in \;𝒫$$ (8)

For *g* we used Fragments Per Kilobase of transcript per Million mapped reads (FPKM) computed by Kuang *et al.* [25] for each the 15 time-points RNA-seq libraries depicted in S1 Fig. To further refine the solution space, we used Kuang *et. al* [25]’s experimentally determined ratios of time-specific oxygen uptake (*w*_*o*2_) to biomass growth rates ($w_{\mu }$). This information was incorporated as the following constraint that ensures our predicted fluxes align with observed physiological parameters.

$$v_{\mu }-\frac{w_{\mu }}{w_{o2}}v_{o2}=0$$ (9)

Thus conditioned, we implemented this transcriptome-aware CBM [42] using the COBRApy 0.22.196 library [55] in Python 3.8.3 [56]. We carried out the non-linear optimization with the IPOPT 3.12.397 optimizer [57] via the CasADi 3.5.598 nonlinear optimization and algorithmic differentiation tool [58]. We deposited all original code at Github (https://github.com/mrivas/epiflux/) and is publicly available as of the date of publication.

## Results

### Metabolism dynamics over the YMC

When grown in continuous culture under a glucose-limiting condition, *S. cerevisiae* exhibits consistent metabolic cycles which have been categorized [25,44,45] as oxidative (OX), reductive building (RB), and reductive charging (RC) phases. Across these phases, dissolved oxygen levels and expression of enzyme-related genes fluctuate significantly [25,44]. Consequently, we expected that the fluxome of *S. cerevisiae* would exhibit a dynamic pattern. To test this, we used RNA-seq data from Kuang *et al*. [25] to infer the fluxome changes using a transcriptome-aware CBM based on the principle of maximum entropy [42]. We further refined the CBM inferences by incorporating constraints on the biomass growth rate and oxygen uptake rates, aligning with the reported values for each phase of the YMC (see Fig 1).

**Fig 2:**
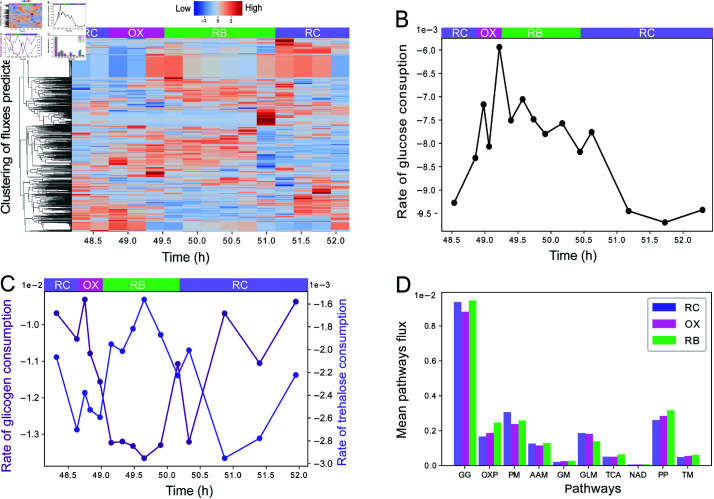
Metabolism dynamics over the YMC. (A) Complete prediction of the distribution of fluxome of *S. cerevisiae* in the YMC, normalized by rows. This dataset is available in tabular format in S1 File. (B) Rate of glucose consumption measured as a fraction of metabolism over the 15 times in the YMC. (C) Rate of trehalose and glycogen consumption in the metabolism, *S. cerevisiae* uses these reserve sugars when glucose is in short supply. In the RC stage, the highest consumption of glucose and trehalose is evident, while glycogen has a higher rate in RB. (D) Enrichment of metabolic pathways in the YMC, the graph is normalized by the sum of all fluxes. Use of various metabolic pathways: gluconeogenesis (GC), oxidative phosphorylation (OXP), pyruvate metabolism (PM), alanine and aspartate metabolism (AAM), glutamate metabolism (GM), glycolipid metabolism (GLM), three carboxylic acid metabolism (TCA), NAD biosynthesis (NAD), pentose phosphate pathway (PP), and transport mitochondrial (TM).

The inferred fluxome dynamic are depicted in Fig 2A, revealing that the vast majority of reactions vary their fluxes over the YMC. Interestingly, their flux clusters are not exclusive to any YMC phase, but rather form complex patterns. For example, the top flux cluster of Fig 2A, shows fluxes peaking at the transition between OX and RB phases, and then again in the RB-RC inter-phase. In general, we did not find any cluster of fluxes being enriched over a single YMC phase for its entire duration, indicating a rather gradual metabolic reprogramming between phases. To gain more insight into this metabolic reprogramming, we explored how these complex fluxome patterns are related to previously reported metabolic traits of *S. cerevisiae*’s YMC.

A perplexing characteristic of *S. cerevisiae*’s YMC is that oxygen and glucose intake rates are not correlated [25,44]. While the highest oxygen intake of *S. cerevisiae* is observed during the OX phase [25,44], the maximum glucose intake occurs during the RC phase [44,59,60]. It is possible that the glucose consumed during the RC phase is stored as glycogen and trehalose, which serve as carbon-source reserves that can be oxidized later during the OX phase [51]. To determine if this is the case, we single out the consumption rates of these three sugars. The consumption rates of glucose (Fig 2B) are consistent with the literature [44,59,60], showing that their magnitude maxes out at the RC phase. In relation to other carbon sources (Fig 2C), we observed that glycogen consumption peaks during the OX and RB phases. This suggests a compensatory mechanism where glycogen consumption complements glucose utilization, particularly when glucose levels diminish. We speculate that this may be a coping mechanism to maintain a continuous intake of carbon source, where the consumption of glucose from the medium during the RC phase is stored as glycogen, which is later consumed during the OX and RB phases. The consumption of trehalose (Fig 2C), on the other hand, seems to play an intermediary role, being consumed primarily at the transition from RC to OX, and then again at the beginning of the RC phase. These results are coherent with the literature [51,61,62].

As aerobic conditions increase the glucose yield in ATP, it would be reasonable to expect that higher oxygen consumption levels coax a surge in metabolic activity. To gauge how the metabolic activity varies between YMC phases, we computed the net flux of oxidative phosphorylation (OXP) by scaling the fluxes by the experimentally derived oxygen fluxes (see Fig 1B). We used OXP as a reference pathway as it is directly related to the oxygen consumption rate, whose wavering behavior defines the YMC. Unsurprisingly, the highest average flux of OXP happens in the OX phase with a net flux of 5.29 (mmol/g/h). On the other hand, the average fluxes of OXP on RB and RC are 5.12 and 4.30, respectively.

Another interesting question is how different stages distribute their metabolic fluxes among different pathways. For this, we analyzed 10 metabolic pathways, quantifying for each of them their average flux magnitude across its reactions (using the reaction pathway membership reported in iMM904) and normalized by the sum of all network flux magnitudes (see Fig 2D). This normalized average flux can be interpreted as the fraction of the metabolic flux that transits over the said pathway. The results show that among RC, OX, and RB phases the pathway carrying most flux is gluconeogenesis (GC). The remaining pathways vary in prominence among YMC phases. For instance, pyruvate metabolism (PM) and glycolipid metabolism (GLM) are more prevalent in RC stage compared to the OX and RB, whereas OXP, pentose phosphate pathway (PP), and transport mitochondrial (TM) dominate at the RB stage compared to the other two stages. No pathway is particularly dominant at the OX stage. This indicates that OX is the phase with the most equally distributed set of fluxes over pathways. Whereas RC and RB concentrate fluxes over specific pathways.

### Acetylation and methylation fluxes peak at different times

Due to their role as cellular regulators of gene expression and metabolic state, the dynamic changes in H3K9Ac [17,18,21] and H4K4me3 [19,20] carry significant importance. These chromatin modifications are sensitive to intracellular levels of acetyl-CoA and SAM [13,25]. By this mechanism, the cellular metabolic state can influence gene expression patterns [29]. Thus, understanding the relationship between H3K9Ac/H3K4me3 and the flux of their epigenetic cosubstrates acetyl-CoA/SAM can provide valuable insights into the molecular regulatory mechanisms of the YMC, as well as the timing of histone marks and transcriptional regulation. To address these questions, we analyzed the flux dynamics of acetyl-CoA and SAM.

**Fig 3:**
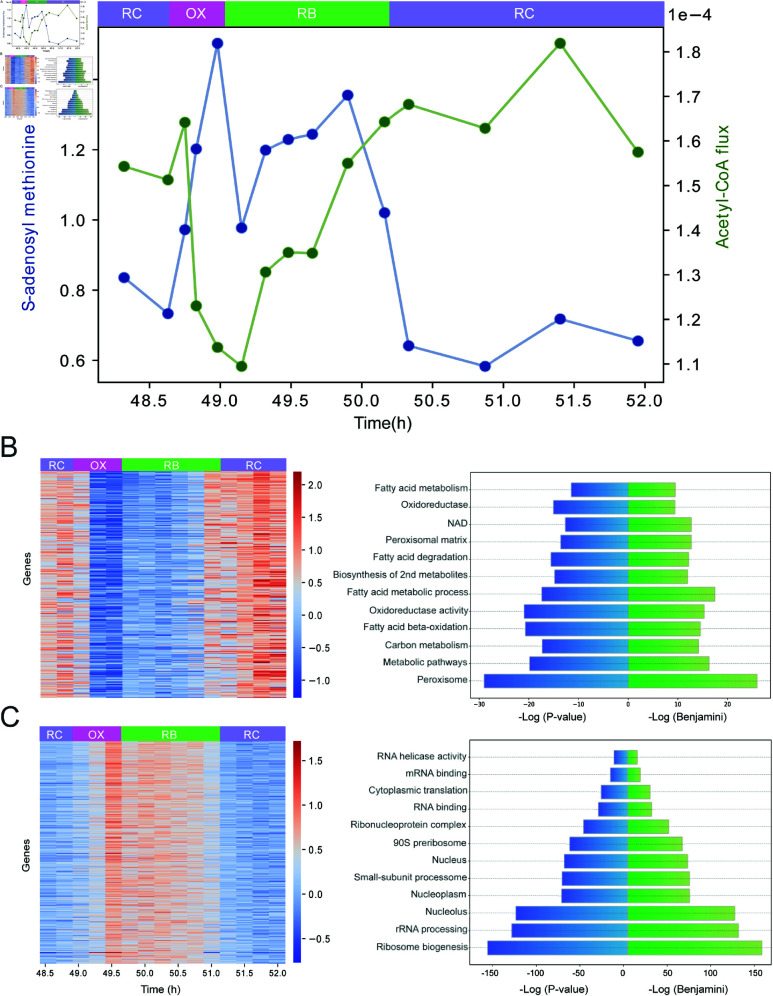
Dynamics of acetylation and methylation in the YMC. (A) Scatter plot of the production fluxes of SAM and acetyl-CoA over 15 sample times. Fluxes are normalized by the sum of all fluxes in the metabolic network. (B) Heat map showing genes (Y-axis) that have a high Pearson correlation (greater than 0.7) between their promoter intensity of H3K9Ac and the flux of acetyl-CoA at various time points. The adjacent figure lists the Gene Ontology terms enriched among these genes (DAVID functional annotation, Benjamini < 0.001). (C) Genes that have a high Pearson correlation (greater than 0.7) between the their promoter intensity of H3K4me3 and the flux of SAM. The adjacent figure lists the Gene Ontology terms enriched among these genes (DAVID functional annotation, Benjamini < 0.001).

Acetyl-CoA flux exhibits a dynamic pattern across the metabolic phases (Fig 3A). Peak flux occurs late in the RC phase and at the transition to the OX phase (RC/OX interface). This is followed by a sharp decrease towards the end of the OX phase, with flux recovery initiating during the RB phase. This pattern is consistent with previously published data on acetyl-CoA levels [45,63], which report peak concentrations immediately following the time points where our model predicts maximum acetyl-CoA flux. Interestingly, results show complementary flux patterns of acetyl-CoA and SAM throughout the YMC (Fig 3A), with the flux of SAM having higher fluxes at OX and RB phases than at RC stage. This suggests that the temporal production patterns of acetyl-CoA and SAM play complementary regulatory roles. To determine the biological functions associated with each epigenetic cosubstrate, we first identified genes whose promoter enrichment of H3K9ac strongly correlated with acetyl-CoA flux profiles (Pearson coefficient ≥ 0.7), and likewise for H3K4me3 and SAM. Subsequently, we used the DAVID Functional Analysis Tool [64,65] to identify statistically significant biological functions (with a Benjamini-corrected p-value < 10^−3^) within these gene sets. Our analysis uncovered that 268 genes linked to acetyl-CoA are primarily involved in metabolic pathways functions (Fig 3B), including peroxisome activity, fatty acid oxidation, glycolysis, and lyase activity (gene names in S2 Fig). On the other hand, 560 genes linked to SAM are mainly involved in cell cycle regulation and protein synthesis (Fig 3C), including ribosome synthesis, nuclear function, and RNA binding, among other key processes (gene names in S3 Fig).

Actively expressed genes are typically enriched at their promoter region by H3K9Ac and H3K4me3 [66]. Thus, we explored whether the flux patterns of acetyl-CoA and SAM translated into increased gene expression by inducing promoter-enrichment of H3K9Ac and H3K4me3, respectively. For this, we analyzed genes categorized by Kuang *et al*. [25] as having peak expressions in the OX (1607 genes), RB (979 genes), or RC (1481 genes) stages. We anticipated that RC-peaking genes (gene expression heatmap S2 FigA) would exhibit a concurrent H3K9Ac enrichment, as this is what the underlying flux pattern of acetyl-CoA would induce. Similarly, we anticipated that H3K4me3 enrichment would mirror the patterns of OX and RB-peaking genes (gene expression heatmaps S3 FigA and S2 FigC), reflecting the flux profile of SAM. Contrary to our expectations, only a subset of genes aligned with these predictions. While some RC genes displayed H3K9Ac enrichment (S2 FigB), only a fraction of OX and RB genes showed H3K4me3 enrichment in their respective peak stages (see S3 FigB and S3 FigD). This discrepancy between gene expression and histone modification enrichment suggests that epigenetic cosubstrate availability alone is insufficient to drive histone PTMs. Therefore, we delve into additional factors influencing the timely enrichment of histone PTMs in the following section.

### There are genes where metabolic and epigenetic states are signficantly correlated

To determine whether the production of epigenetic co-substrates affects histone mark enrichment, we calculated the correlation between the temporal profiles of gene promoter enrichment for H3K9ac/H3K4me3 and the production fluxes of acetyl-CoA/SAM. These correlation values served as indicators of metabolic dominance over epigenetic state, which we will refer to as metabolic dominance for brevity.

To address potential spurious correlations, we constructed a null distribution where metabolic dominances were inherently random. Specifically, for each gene, we randomly shuffled the time series of both acetyl-CoA production fluxes and H3K9ac levels, and then calculated their correlation. We repeated this process for the temporal profiles of SAM versus H3K4me3.

**Fig 4:**
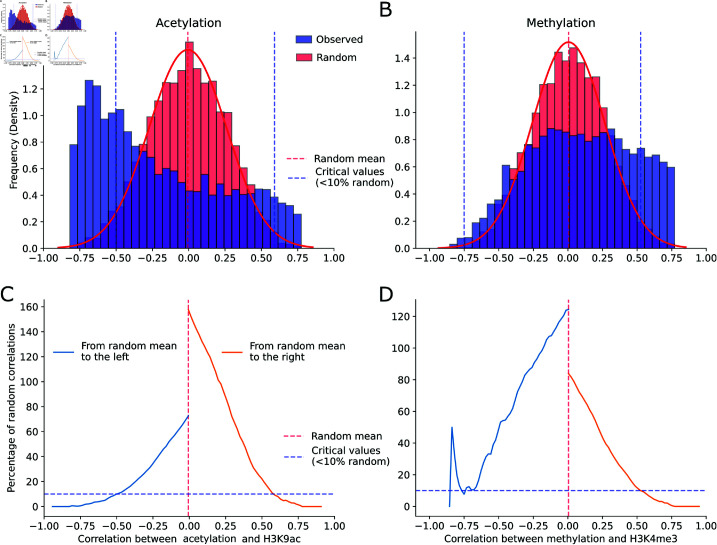
Statistical assessment of metabolic dominance. Distributions of random and observed metabolic dominances, measured as correlations between the temporal profiles of promoter enrichment of histone marks and the flux patterns of their co-substrates fluxes. Results are presented for (A) acetylation H3K9Ac/acetyl-Coa, and (B) metylation H3K4me3/SAM. Percentage of random to observed correlations (y-axis) at various critcal values (x-axis) for (C) acetylation and (D) methylation.

For acetylation co-substrates (Fig 4A), the observed metabolic dominance values significantly deviated from the null distribution, producing heavier tails compared to the random distribution. To assess the statistical significance of a metabolic dominance value, we calculated the ratio of random to observed genes with equal or more extreme values (Fig 4C). We found that metabolic dominances above 0.591 contain less than 10% of random correlations (33 out of 366 genes). Similarly, metabolic dominances below -0.502 contain less than 10% of random correlations (178 out of 1799 genes).

In contrast, for methylation co-substrates (Fig 4B), only positive correlation values were significantly observed. Specifically, metabolic dominances above 0.525 were observed to have less than 10% of random correlations (Fig 4D). This means that, out of 1064 genes, no more than 106 correlations originated from the random distribution.

The observed correlation between co-substrate dynamics and histone modifications may be mediated by chromatin accessibility, as suggested by previous studies [30,31]. This mechanism is further explored in the next section.

### Chromatin accessible states precede histone modifications

We investigated whether chromatin accessibility precedes histone modifications in the yeast metabolic cycle (YMC), as suggested by previous research [30,31]. To study this relationship, first we utilized ATAC-seq data from Gowans *et al*. [43] to evaluate chromatin accessibility across six time points (OX I, OX II, RB I, RB II, RC I, and RC II) during the yeast metabolic cycle (YMC). This quantification allows us to show dynamic changes in chromatin accessibility at the promoter region for each gene throughout the cycle. Second, we assess the influence of the metabolic state on genes’ epigenetic state by their metabolic dominance.

**Fig 5:**
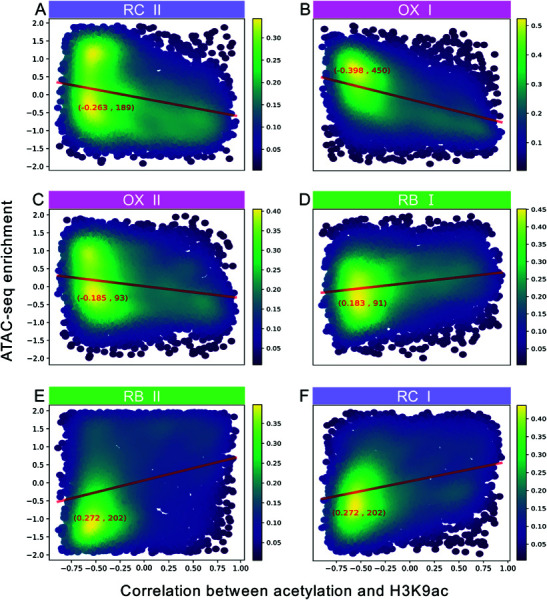
Chromatin accessibility at genes sorted by their correlation between enrichment of H3K9Ac and production flux of acetyl-CoA. The multipanel shows the accessibility of chromatin at 6 sample times of ATAC-seq spanning all the YMC stages. Sequential samples at early and late OX phase are denoted as OX I and OX II, and likewise for the RB (RB I and RB II) and RC phases (RC I and RC II). The x-axis present genes sorted by their correlations between their temporal profiles of H3K9ac promoter enrichment and acetyl-CoA production flux. The y-axis show the gene promoter enrichment of ATAC-seq. Red lines indicate linear correlation, with values in parenthesis presenting: Pearson correlation value, and -log(P-value).

**Fig 6:**
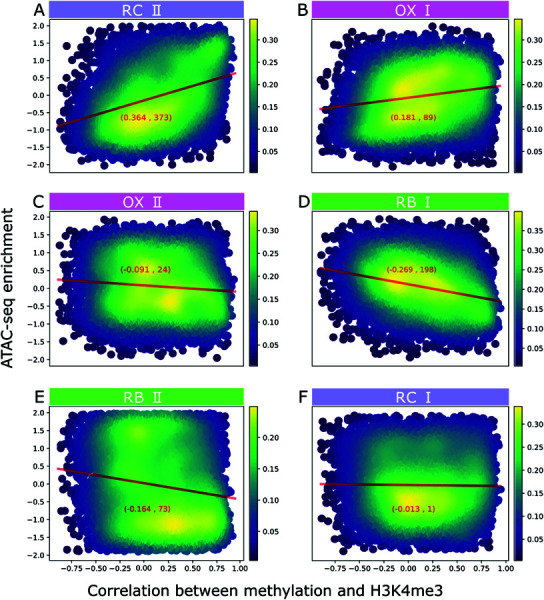
Chromatin accessibility at genes sorted by their correlation between enrichment of H3K4me3 and production flux of SAM. The multipanel shows the accessibility of chromatin at 6 sample times of ATAC-seq spanning all the YMC stages. Sequential samples at early and late OX phase are denoted as OX I and OX II, and likewise for the RB (RB I and RB II) and RC phases (RC I and RC II). The x-axis present genes sorted by their correlations between their temporal profiles of H3K4me3 promoter enrichment and SAM production flux. The y-axis show the gene promoter enrichment of ATAC-seq. Red lines indicate linear correlation, with values in parenthesis presenting: Pearson correlation value, and -log(P-value).

We created density plots in Figs 5 and 6 for each time point where genes are arranged by metabolic dominance on the x-axis and ATAC-seq enrichment on the y-axis. These figures indicate a potential sequence in chromatin dynamics where the highest production flux of acetylation and methylation happen at times preceded by states of chromatin opening. Specifically, an increase relationship between ATAC-seq signal and metabolic dominance was noted in the RB I and II sample points (Fig 5D and 5E show positive and statistically significant Pearson correlations), indicating chromatin opening precedes the acetylation process. This suggests acetyltransferases may utilize cytoplasmic acetyl-CoA for H3K9ac marking subsequent to chromatin opening. Likewise, for methylation, chromatin opening initiates in the RC II sample point (Fig 6A showing positive and statistically significant Pearson correlation between ATAC-seq signal and metabolically dominated genes), potentially leading to a stronger correlation between SAM flux and H3K4me3 in subsequent stages.

Thus, our analysis of chromatin accessibility and acetyl-CoA/SAM flux predictions supports the idea of a sequential process, where chromatin opening is a prerequisite for subsequent histone acetylation or methylation, utilizing available cosubstrates in the cytoplasm.

## Discussion

In this study, we addressed the interplay between metabolic production of epigenetic cosubstrates and histone PTMs. Our findings provide novel insights into the dynamic interplay between metabolism and epigenetic modifications, highlighting the importance of considering multiple cosubstrates and chromatin accessibility in understanding epigenetic regulation. We focused on the production fluxes of acetyl-CoA and SAM and their impact on epigenetic marks H3K9Ac and H3K4me3. We integrated transcriptomic and fluxomic data to calculate these fluxes, with the estimated glucose consumption data (Fig 2B) being consistent with the literature [44,59,60]. Our findings reveal a significant correlation between these metabolic fluxes and specific biological functions: acetyl-CoA flux was correlated with genes involved in metabolic functions, while SAM flux was correlated with genes involved in protein synthesis and cell cycle regulation, which is consistent with the literature [44,59,60].

Furthermore, ATAC-seq data analysis suggests chromatin accessibility is a prerequisite for histone acetylation and methylation. This discovery highlights the nuanced relationship between metabolic fluxes and epigenetic modifications, influenced by chromatin states.

Overall, our results support the hypothesis of metabolic-epigenetic interplay and contribute to understanding the regulatory mechanisms in *S. cerevisiae* YMC, offering insights into broader biological processes like cell differentiation and cancer transformation. However, it must be noted that our results may be affected by the source data we used to condition our estimation of metabolic fluxes. In particular, gene expression is an often far-from-perfect quantification of the amount of a protein available for a reaction. Firstly, protein expression is regulated at both the transcriptional and translational levels, so gene expression is often not indicative of protein expression. Secondly, proteins often have multiple functions, so much of the protein in a cell might be unavailable for catalyzing a specific reaction. To overcome this limitation, a direct quantification of enzymes concentrations could improve the fluxes estimations. Additionally, we have concocted data of chromatin openness (ATAC-seq), on one hand, and gene expression and histome PTMs, on the other, from two different labs, which may have inherited lab-related differences in preparation protocols which may present biased our results. Promoters for genes that are not expressed are sometimes open or in H3K4me3 or H3K9ac regions. Thus, promoter openness or marking by a promoter-associated histone modification is not always indicative of expression of the corresponding gene. Finally, it is important to acknowledge that the intricate dynamics between metabolic and epigenomic states cannot be fully captured by the current dataset. Future research seeking a more nuanced understanding of these dynamics could benefit from incorporating the influence of transcription factors [67–69] and the temporal expression patterns of chromatin-modifying enzymes [70,71] on histone modification dynamics.

## Supporting information

S1 Fig Time points sampled during the YMC. The original time points sampled for RNA-Seq data (A. top) and ChIP-Seq data (A. bottom) at each YMC phase are shown. On the left panel, the 15-time points after the alignment of the two-time series are displayed: time points 10 and 11 from RNA-seq were averaged, as well as time points 13 and 14 from ChIP-seq. On the right panel Y axis the percentage of oxygen in the environment is indicated. Figure adapted from Sanchez *et al*. [18].
(TIFF)

S2 Fig Genetic and epigenetic profiles at RC stage during the YMC. (A) Expression heatmap of genes reported in literature [25] as being characteristic of the RC stage. (B) Heatmap of promoter enrichment of H3K9Ac at RC| stage. Genes are sorted by their correlation between gene expression and acetyl-CoA flux profiles. Highly correlated genes at the top.
(TIFF)

S3 Fig Genetic and epigenetic profiles at OX and RB stages during the YMC. (A) Expression heatmap of genes reported in literature [25] as being characteristic of the OX stage. (B) Heatmap of promoter enrichment of H3K4me3 at OX stage. Genes are sorted by their correlation between gene expression and SAM flux profiles. Highly correlated genes at the top. (C) Expression heatmap of genes reported in literature [25] as being characteristic of the RB stage. (D) Heatmap of promoter enrichment of H3K4me3 at RB stage. Genes are sorted by their correlation between gene expression and SAM flux profiles. Highly correlated genes at the top.
(TIFF)

S1 Table Reactions associated with acetylation. These reactions are mediated by the enzyme ATP citrate lyase [40].
(PDF)

S2 Table Reactions associated with methylation. These reactions are mediated by the methionine cycle.
(PDF)

S1 File Normalized flues over the YMC. These are the fluxes used for Fig 2A.
(XLSX)

S2 File Genes that have a high Pearson correlation between their promoter intensity of H3K9Ac and the flux of acetyl-CoA These are the fluxes used for the heatmap in Fig 3B.
(XLSX)

S3 File Genes that have a high Pearson correlation etween the their promoter intensity of H3K4me3 and the flux of SAM These are the fluxes used for the heatmap in Fig 3C.
(XLSX)
