# Supplementary figures and images for: Dynamic metabolic regulation of histone modifications during the yeast metabolic cycle
Source: PLoS One. 2025 May 20;20(5):e0323242. doi: 10.1371/journal.pone.0323242 (PMC12091797; doi:10.1371/journal.pone.0323242)

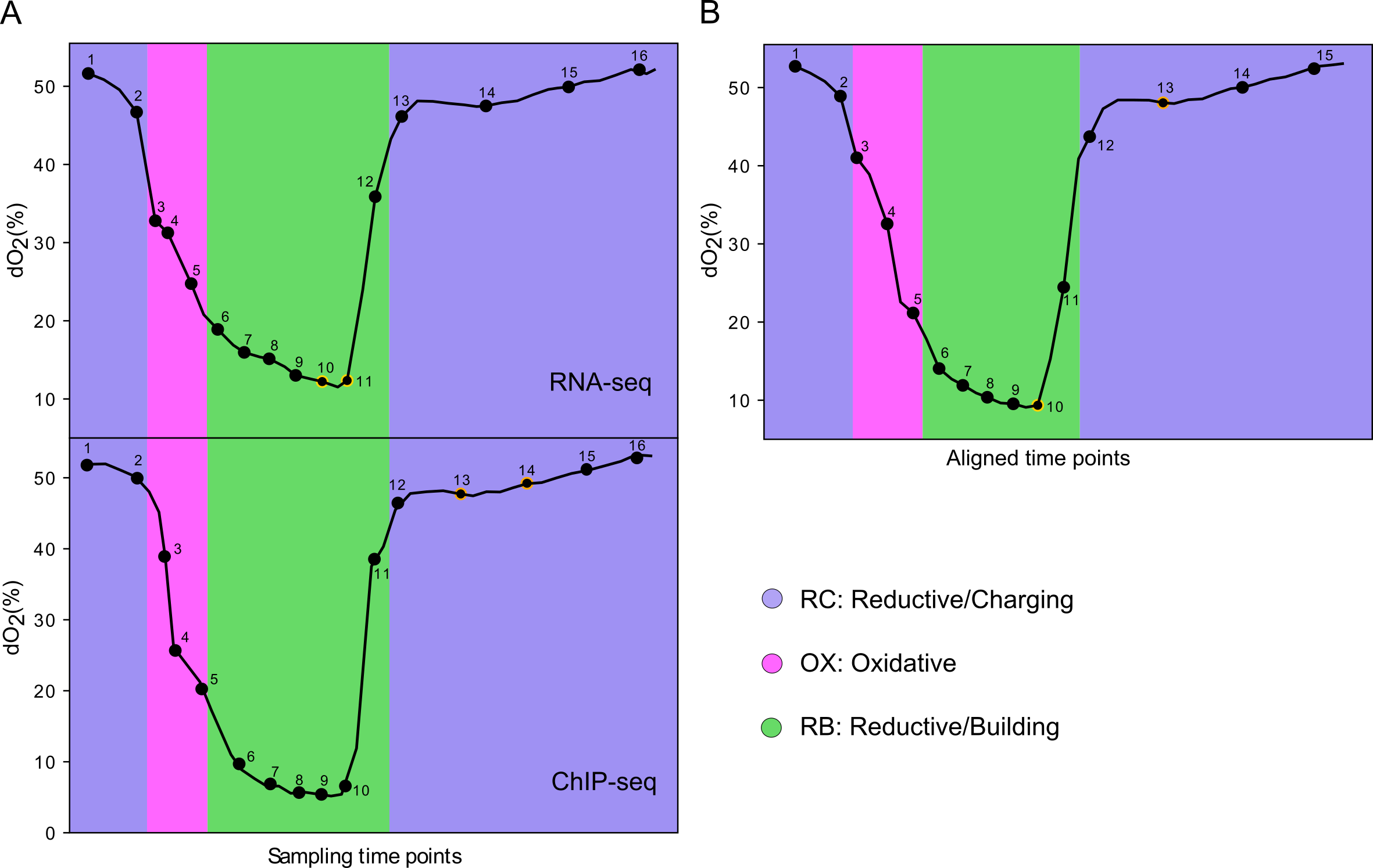

Supplement: S1 Fig — The original time points sampled for RNA-Seq data (A. top) and ChIP-Seq data (A. bottom) at each YMC phase are shown. On the left panel, the 15-time points after the alignment of the two-time series are displayed: time points 10 and 11 from RNA-seq were averaged, as well as time points 13 and 14 from ChIP-seq. On the right panel Y axis the percentage of oxygen in the environment is indicated. Figure adapted from Sanchez et al. [18]. (TIFF) [file pone.0323242.s001.tif]

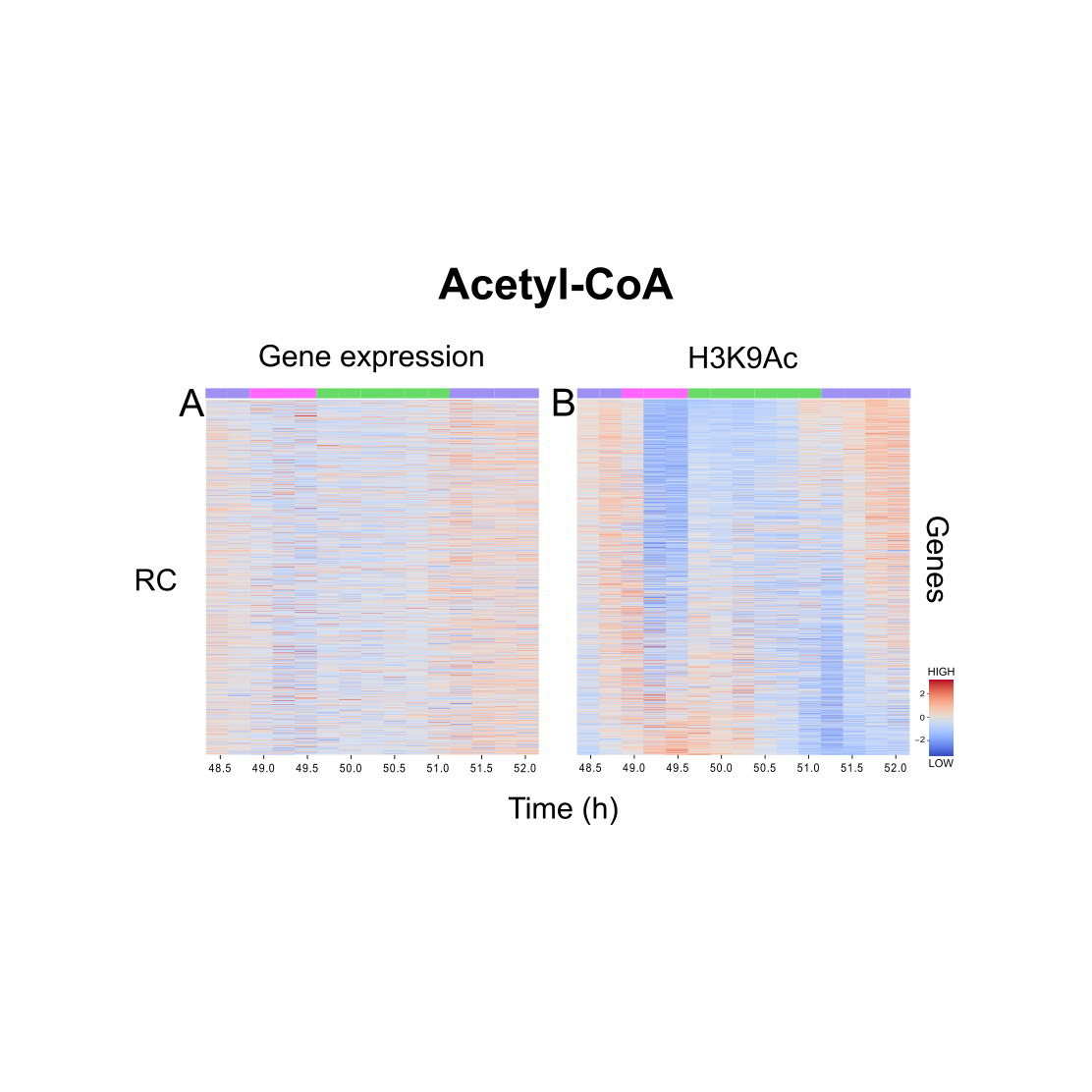

Supplement: S2 Fig — (A) Expression heatmap of genes reported in literature [25] as being characteristic of the RC stage. (B) Heatmap of promoter enrichment of H3K9Ac at RC| stage. Genes are sorted by their correlation between gene expression and acetyl-CoA flux profiles. Highly correlated genes at the top. (TIFF) [file pone.0323242.s002.tif]

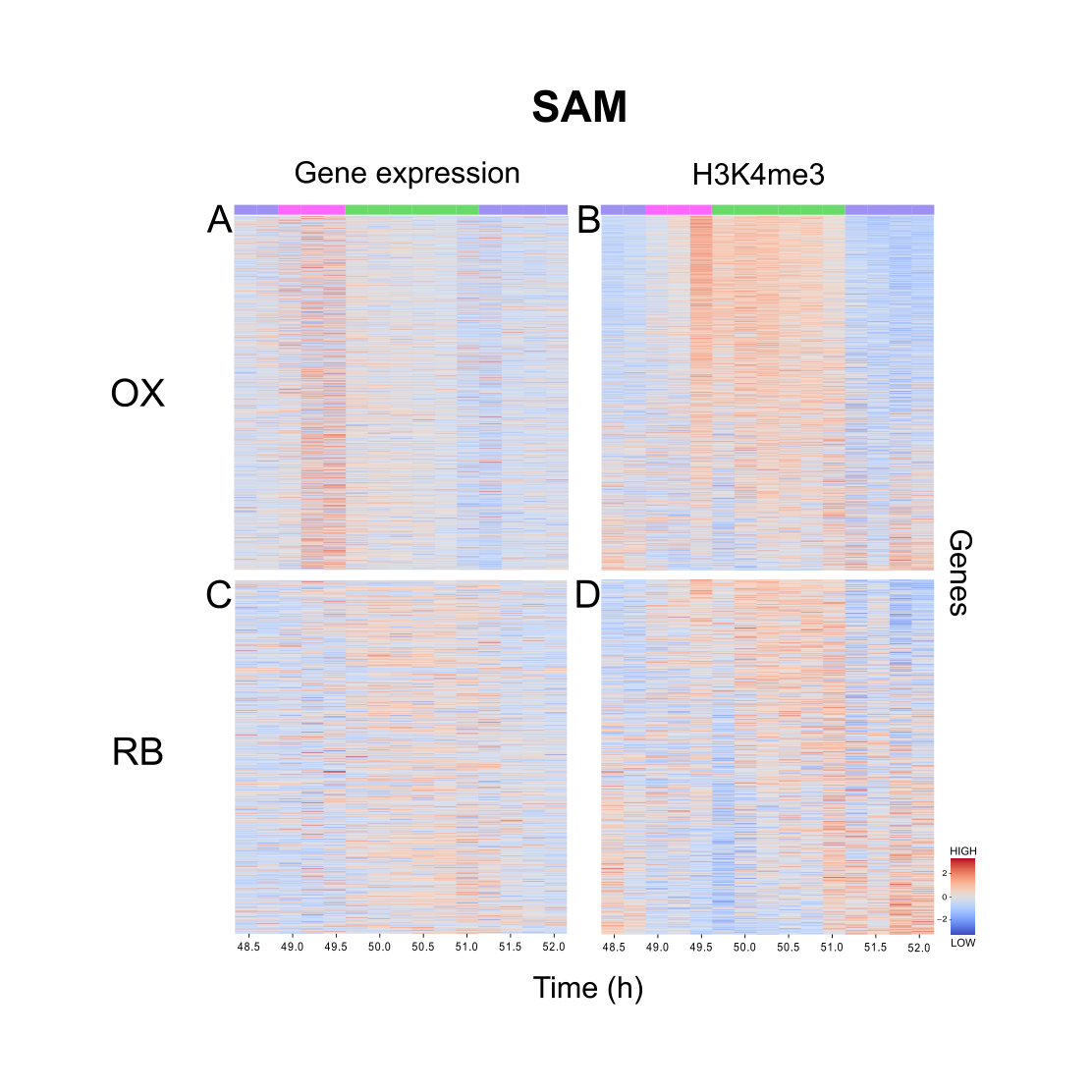

Supplement: S3 Fig — (A) Expression heatmap of genes reported in literature [25] as being characteristic of the OX stage. (B) Heatmap of promoter enrichment of H3K4me3 at OX stage. Genes are sorted by their correlation between gene expression and SAM flux profiles. Highly correlated genes at the top. (C) Expression heatmap of genes reported in literature [25] as being characteristic of the RB stage. (D) Heatmap of promoter enrichment of H3K4me3 at RB stage. Genes are sorted by their correlation between gene expression and SAM flux profiles. Highly correlated genes at the top. (TIFF) [file pone.0323242.s003.tif]
