# Supplementary material for: Dynamic metabolic regulation of histone modifications during the yeast metabolic cycle
Source: PLoS One. 2025 May 20;20(5):e0323242. doi: 10.1371/journal.pone.0323242 (PMC12091797; doi:10.1371/journal.pone.0323242)
Supplement: S1 Table — These reactions are mediated by the enzyme ATP citrate lyase [40]. (PDF) [file pone.0323242.s004.pdf]

Reaction Name	Reaction
Nuclear citrate	$cit\_n \rightleftharpoons cit\_c$
Nuclear oxalacetate	$oaa\_n \rightleftharpoons oaa\_c$
Acetyl-CoA	$atp\_n + cit\_n + coa\_n \rightarrow accoa\_n + adp\_n + oaa\_n + pi\_n$
Protein + Acetyl-CoA	$accoa\_n + prot\_n \rightarrow coa\_n + prot\_ac\_n$
Protein exchange	$\rightarrow prot\_n$
Protein acetylated exchange	$prot\_ac\_n \rightarrow$
