# Supplementary material for: Dynamic metabolic regulation of histone modifications during the yeast metabolic cycle
Source: PLoS One. 2025 May 20;20(5):e0323242. doi: 10.1371/journal.pone.0323242 (PMC12091797; doi:10.1371/journal.pone.0323242)
Supplement: S2 Table — These reactions are mediated by the methionine cycle. (PDF) [file pone.0323242.s005.pdf]

Reaction Name	Reaction
Alfa-ketoglutarate - Succinate	$akg\_n + o2\_n + s\_n \rightleftharpoons succ\_n + co2\_n + so2\_n$
Alfa-ketoglutarate	$akg\_n \rightleftharpoons akg\_c$
Alfa-ketoglutarate reductase; NAD-dependent	$nad\_n + 2hglut\_n \rightleftharpoons akg\_n + h\_n + nadh\_n$
Succinate	$succ\_n \rightleftharpoons succ\_c$
Nuclear homocysteine	$hcys\_\_L\_n \rightleftharpoons hcys\_\_L\_c$
S-adenosyl-L-homocystine	$ahcys\_n \rightleftharpoons ahcys\_c$
S-adenosyl-L-methionine	$amet\_c \rightleftharpoons amet\_n$
SAM	$amet\_n + hcys\_\_L\_n \rightarrow ahcys\_n$
Protein + SAM	$amet\_n + hcys\_\_L\_n + prot\_n \rightarrow ahcys\_n + h\_n + prot\_me\_n$
Protein methylated exchange	$prot\_me\_n \rightarrow$
